# What makes joint assessment procedures attractive to the innovative industry: successes, challenges, and proposed improvements

**DOI:** 10.3389/fmed.2023.1207954

**Published:** 2023-09-04

**Authors:** Nevena Miletic, Sarah Adam, Jacqueline Acquah, Zainab Aziz, Angelika Joos, John M. Mwangi

**Affiliations:** ^1^F. Hoffmann–La Roche Ltd, Basel, Switzerland; ^2^The International Federation of Pharmaceutical Manufacturers and Associations, Geneva, Switzerland; ^3^Johnson & Johnson Middle East FZ-LLC, Accra, Ghana; ^4^Novartis South Africa (Pty) Ltd, Magwa Crescent West, Johannesburg, South Africa; ^5^MSD (Europe) Inc., Brussels, Belgium; ^6^Bayer Pharmaceuticals, Nairobi, Kenya

**Keywords:** reliance, regulatory convergence, collaboration, regulatory agilities, African Medicines Agency, African Medicines Regulatory Harmonization, joint assessment procedures

## Abstract

Regulatory harmonization and convergence have been identified as the key driver in promoting efficient evaluation of medicines, reducing workload, and supporting earlier access to medicines on the African continent. There has been great progress to date in enhancing regulatory harmonization and convergence on the African continent via the Regional Economic Communities (RECs) and with the establishment of the Africa Medicines Agency (AMA). In this article, the International Federation of Pharmaceutical Manufacturers and Associations (IFPMA) Africa Regulatory Network (ARN) presents its perspective based on the available literature review and results from a survey conducted with innovative biopharmaceutical companies to gather experiences using regional joint assessment procedures (JAPs) in Africa, such as the East African Community Medicines Regulatory Harmonization (EAC-MRH), the West African Medicines Regulatory Harmonization (WA-MRH), and the Southern African Development Community Medicines Regulatory Harmonization (SADC-MRH) initiative through the ZAZIBONA Collaborative Procedure for Medicines Registration (ZaZiBoNa), and provides best practices in this evolving landscape. The article also assesses other collaborative registration pathways available to facilitating registration of pharmaceutical products in African countries, such as WHO Collaborative Registration Procedures (CRP), Swissmedic’s Marketing Authorisation for Global Health Products (MAGHP) and EU Medicines for All (EU-M4ALL). Benefits and challenges of each of the existing pathways are discussed in this article. Main benefits include building more expert capacity and improved collaboration amongst experts, as well as shorter review timelines in some cases. Key challenges include the lack of predictability in the adherence to procedural timelines as defined per guidelines, lengthy timeline to achieve national marketing authorization following joint assessment, the lack of dedicated personnel, administrative issues during the submission process as well as additional country-specific requirements on top of JAP-specific requirements. Our recommendations for improvements include harmonization of requirements across countries and regions and with international standards, appropriate resource allocation for JAP activities to ensure adherence to timelines, use of JAPs throughout the entire product lifecycle and all product categories, adequate use of digital technologies, and improved communication and transparency with applicants. These improvements will allow industry to better plan their filing strategies for the region which will lead to overall improved usability of the JAPs in Africa and enable faster patient access.

## 1. Introduction

The African Medicines Regulatory Harmonization (AMRH) initiative was established in 2009 to strengthen regulatory systems across national, regional, and continental levels. Leveraging a decade of harmonization activities on the continent and the learnings gained during the pandemic, the initiative is working towards establishing the African Medicines Agency (AMA). The AMA’s objective (1) is to enable efficient and streamlined use of resources to enable scientific regulatory decisions, minimize administrative hurdles, increase reliance based on harmonized regulatory requirements, and improve work sharing synergies and collaboration. The aim is to ensure overall optimization of the healthcare system and timely access to effective, safe, and quality medicines for patients.

The progress made to date with harmonization procedures and convergence (2) provides an optimistic forecast for medication access throughout Africa. Many national regulatory agencies (NRAs) are also developing efficient reliance pathways, taking assessments from products already approved by stringent regulatory authorities (SRAs) into account to speed up their decision making.

Regional economic communities (RECs) in Africa are also developing regional harmonization efforts, resulting in the publication of harmonized regional regulatory guidelines and establishment of regional collaborative and work-sharing procedures, so called joint assessment procedures (JAPs). Table 1 provides an overview of the three regional JAPs we are focusing on in this article: the East African Community Medicines Regulatory Harmonization (EAC-MRH), the West Africa Medicines Regulatory Harmonization Project (WA-MRH), and ZaZiBoNa.

**Table 1:** Joint assessment procedures overview.

Characteristics	EAC-MRH	WA-MRH	ZaZiBoNa
Participating countries	Burundi, Kenya, Rwanda, South Sudan, Tanzania, Uganda	Benin, Burkina Faso, Cabo Verde, Côte d’Ivoire, The Gambia, Ghana, Guinea, Guinea-Bissau, Liberia, Mali, Niger, Nigeria, Senegal, Sierra Leone, Togo	*Active*: Botswana, Democratic Republic of the Congo, Malawi, Mozambique, Namibia, South Africa, Tanzania, Zimbabwe, Zambia *Non-active*: Angola, Comoros Islands, Madagascar, Seychelles, Swaziland *Observers*: Lesotho, Mauritius
Product eligibility	Common mapped applications already submitted to at least two NRAs, biotherapeutics, and biosimilars. Interested applicants are invited to submit applications for all medicinal products; however, the priority will be given to the following^a^: I. Priority list medicines for management of the following medical conditions: Maternal, neonatal, and children’s health-related medical conditions HIV, malaria, tuberculosis, reproductive and neurological disorders Neglected diseases: leishmaniasis, pneumocytosis, toxoplasmosis, filariasis, strongyloidiasis Cancer, diabetes, hypertension. Kidney conditions, hepatic conditions, neurological conditions II. Prescription medicines from domestic manufacturers within the EAC region III. Biotherapeutic products and biosimilars	Based on EOI published by ECOWAS Secretariat^b^ Products on WHO Model List of Essential Medicines Programme Medicines (HIV/AIDS, malaria, tuberculosis, reproductive health, neglected tropical diseases and antibiotics) Medicines used in public health emergencies Products registered by SRAs, prequalified by WHO, and registered under Swissmedic MAGHP Procedure or EMA Article 58 (positive scientific opinion) LSC by the UN Commission on Life-Serving Commodities for Women and Children (3) Biological products (including vaccines) Blood products Medical devices on a WAHO specific list to be published in the EOI Other priority medical products that WAHO will determine from time to time	All essential medicines and medicines used in the treatment of the SADC priority diseases or conditions (4): HIV/AIDS Tuberculosis Malaria Acute respiratory infections Diarrhoea Diabetes Pneumonia Cardiovascular Cancer Obstetrics Gastroenteritis and colic Reproductive health products Products included in the UN Commission for Life-Saving Commodities for Women and Children (3)
Scope of the procedure	EAC Joint Regulatory Review (5): Evaluation of product dossiers Joint inspection of manufacturing sites/desk review, according to the EAC Guidelines on GMP Joint inspections of clinical sites (if applicable), according to the GCP Joint post-marketing quality surveillance and safety reporting Enforcement of regulatory decisions	Joint Medicines Dossier Evaluation Procedure Classes of Applications (6): New applications Renewal of applications (i.e., registration) Variation of applications (i.e., of a registered product)	Joint GMP inspections Collaborative assessment of NDA
Timelines	181 working days of the regulator’s time and at least 180 calendar days applicant’s time with a maximum of three query cycles (5) 300 days for joint assessment and up to 3 months for NRAs to issue MA (7)	The duration of the process^b^ from submission of the application to the final committee recommendation should take ~133 calendar days (for a complete dossier that receives no queries) and 196 calendar days with a single round of questions The national competent authority delivers the MA within a maximum of 60 days after the applicant has filed with the NRA the specific product, the WAHO notification of recommendation, and the relevant local requirements (including national fees)	Up to 9 months for a joint assessment (4) Up to 90 days for individual NRAs adoption of joint assessment recommendation and issuing of national MA (4)
Requirements	Cover letter (8) EAC CTD Samples (2+) NRA processing fee During product evaluation, the NRA may request for further information and additional supporting documents from the applicant		Cover letter Application fees, any statutory forms and product samples with labeling in compliance with individual country requirements Product dossier in SADC CTD format (country-specific Module 1 and identical Modules 2–5) (9)

AIDS, acquired immunodeficiency syndrome; CTD, Common Technical Document; EAC, East African Community; EAC-MRH, East African Community Medicines Regulatory Harmonization; ECOWAS, Economic Community of West African States; WA-MRH, West Africa Medicines Regulatory Harmonization; EMA, European Medicines Agency; EOI, expression of interest; GCP, Good Clinical Practice; GMP, Good Manufacturing Practice; HIV, human immunodeficiency virus; LSC, life-saving commodities; MA, Marketing Authorization; MAGHP, Marketing Authorisation for Global Health Products; NDA, New Drug Application; NRA, national medicines regulatory authority; SADC, Southern African Development Community; SRA, stringent regulatory authorities; UN, United Nations; WAHO, West African Health Organization; WHO, World Health Organization.

a Based on EOI published by EAC Secretariat.

b Economic Community Of West African States (ECOWAS), West Africa Medicines Regulatory Harmonization (WA-MRH) Joint Assessment Procedure For Medicine Registration And Marketing Authorization Of Medicinal Products, September 2022.

Institutions, such as the World Health Organization (WHO), in addition to well-established regulatory authorities, such as the European Medicines Agency (EMA) and Swissmedic, are supporting less-resourced NRAs by performing assessments to assist in the approval of medicinal products in Africa. Examples of assessment programs include:

- WHO Collaborative Registration Procedure for Pre-Qualified Products (WHO PQ CRP).
- WHO Collaborative Registration Procedure for Stringent Regulatory Authority approved products (WHO SRA CRP).
- EU-Medicines for all or ‘EU-M4ALL’.
- Swissmedic Marketing Authorisation for Global Health Products (MAGHP) and MAGHP Light Procedure.

**Figure 1:**
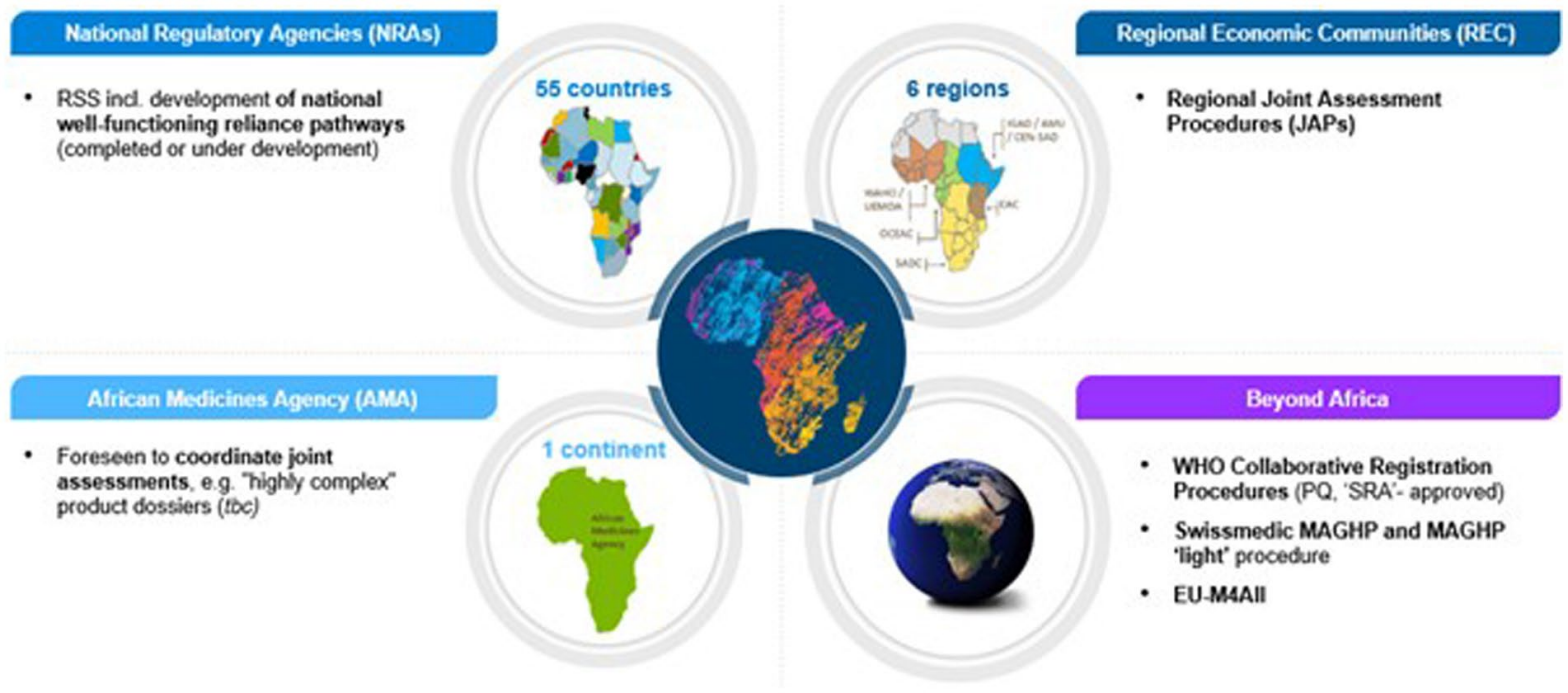
Overview of national, regional, continental and global regulatory procedures applicants can use for medicines registration in Africa.

Considering the number of regulatory procedures industry applicants can use when bringing new medicines to Africa, it is important to have a clear understanding of each program, its advantages and limitations, as well as how they are related to each other in order to determine the optimal filing strategy. Figure 1 details the various harmonization and JAP programs used in Africa.

**Figure 2:**
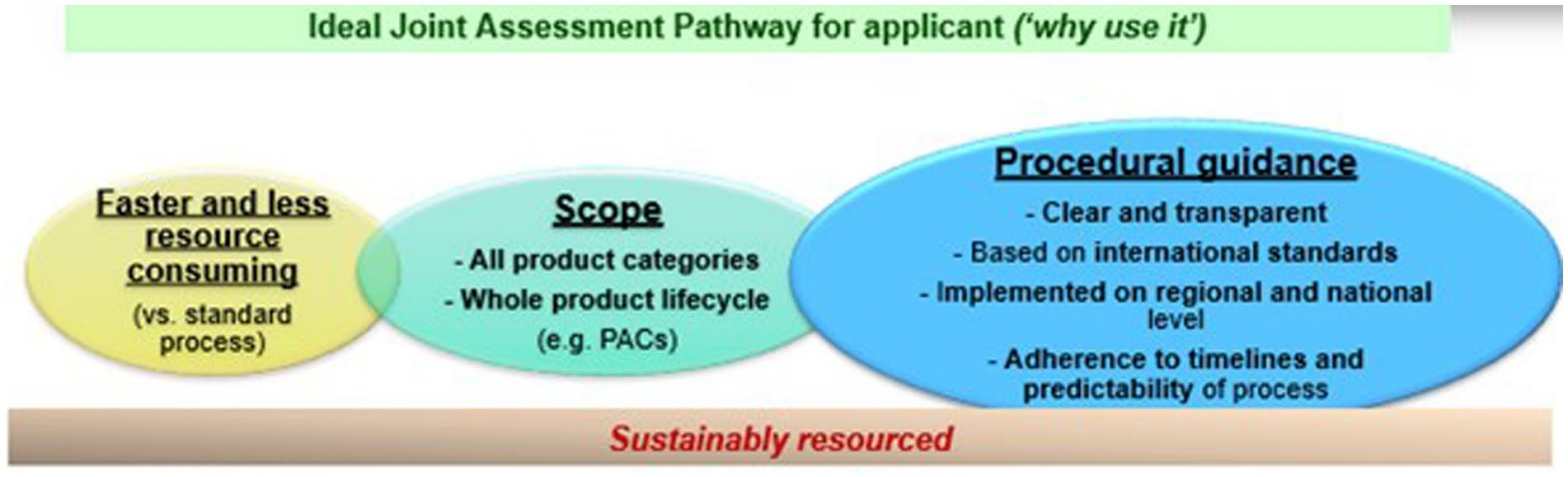
Key considerations for regulatory assessment pathway to be utilized by applicants.

Multiple criteria are important for industry to utilize a joint assessment pathway (see Figure 2). A JAP process must be faster and less resource-consuming than the standard pathway. The scope of a JAP must also allow the industry to utilize the procedure for different product modalities and throughout their product lifecycle. Finally, a JAP procedure must offer clear and transparent procedural guidance, based on international standards, that are implemented in the national procedures and regulations, to make the process attractive and predictable for the industry. Unfortunately, there is not much guidance for the industry on the advantages of using one procedure versus another.

There have been review articles providing perspectives on the various work-sharing and collaborative registration procedures in Africa, both from an industry and a regulator’s standpoint. In addition, surveys and general feedback discussions have continued to report positive experiences (7, 10), including:

- Building the expert capacity of member countries, information sharing, and collaboration among national regulators, leading to improvements in how agencies perform regulatory reviews in their respective countries (11)
- Shorter timelines for approval of medicinal products, resulting in quicker access and increased availability of medicines and vaccines for patients in the region.

Opportunities identified for improvement within the review articles include (2):

- Centralization of submission and efficient tracking system
- Utilization of integrated information management system
- Transparency about the procedures
- Challenges in monitoring and tracking regulators’ assessment reports
- Inadequate funding and human resources (12)
- Manufacturers’ failure to submit the exact same dossier to all countries of interest.

In this article, the IFPMA ARN presents its perspective based on the available literature review, results from a survey conducted with innovative biopharmaceutical companies to gather experiences from using regional JAPs in Africa and provides best practices in this evolving landscape.

## 2. Assessment of policy/guideline options and implications

### 2.1. Methods

Data was collected from literature review and surveys conducted among IFPMA ARN member companies and associations.

#### 2.1.1. Literature search

A literature search was performed during the second half of 2022 in PubMed using following key words/phrases: “East Africa Community,” “joint assessment,” “ECOWAS,” “WA-MRH,” “ZAZIBONA,” “AMRH.” The focus was places on articles published from 2019 to 2022, to correlate with the period when ARN started its analysis work of the JAPs, including industry experience gathering and conducting the industry survey among IFPMA ARN member companies.

Information was also obtained from official regional JAPs or REC websites, published regional guidelines, and quantitative data presented in the public regional events between 2019 and 2022 by representatives of REC secretariats.

#### 2.1.2. Industry survey

The survey was conducted between June and July of 2022 among IFPMA ARN member companies and associations using four Survey Monkey developed questionnaires. Three questionnaires focused on experiences with one of the selected JAPs (EAC-MRH, WA-MRH, ZaZiBoNa) and one focused on experiences with global procedures applicable for supporting marketing authorization (MA) approval in African countries (i.e., WHO CRP PQ, WHO CRP SRA, EU-M4ALL, MAGHP, and MAGHP Light). A total of 12 companies provided responses, each registering between one and fifteen products through the JAPs.

**Figure 3:**
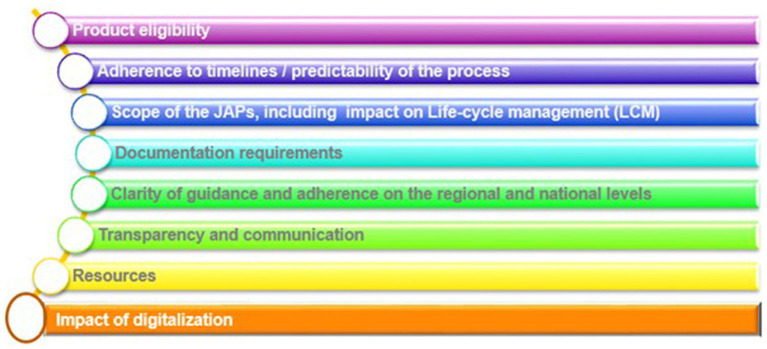
Overview of priority areas assessed in this article.

Data was collected to focus on eight predefined priority areas, as shown in Figure 3.

### 2.2. Results and discussion

#### 2.2.1. Product eligibility

**Figure 4:**
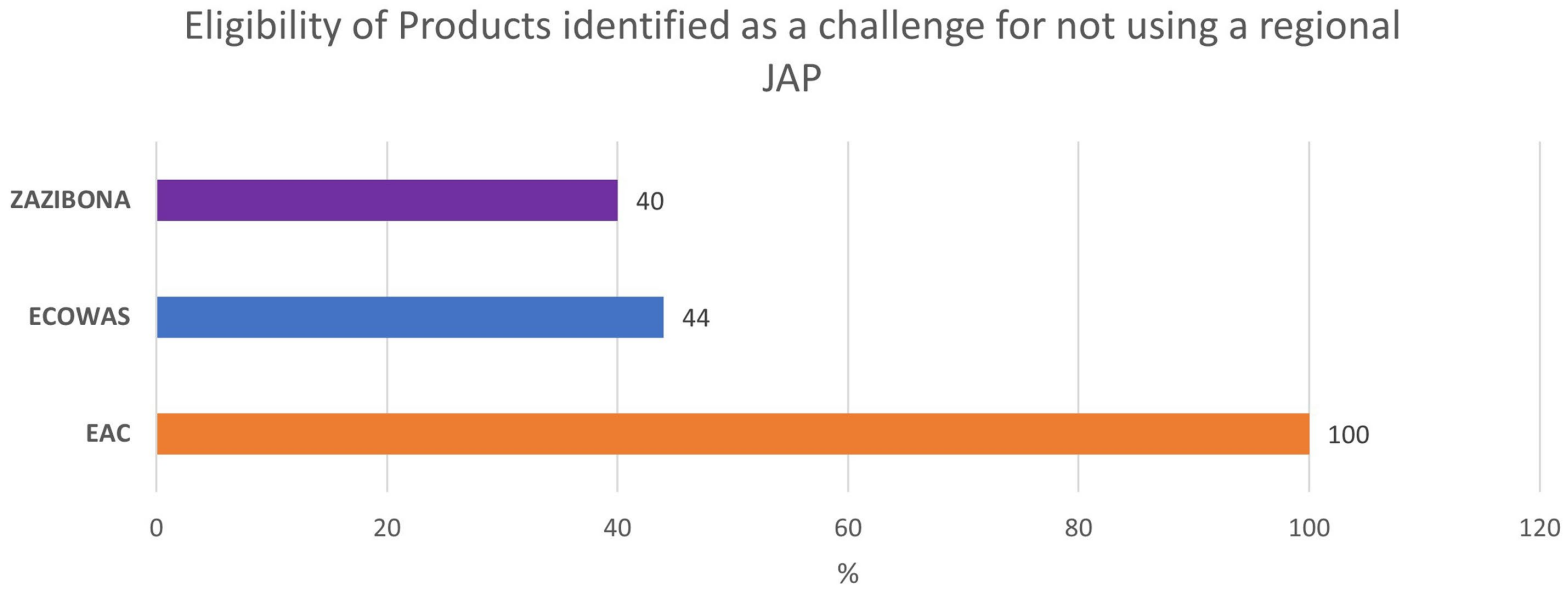
Industry feedback on identifying *Eligibility of Products* as a challenge for not using a specific JAP.

Product eligibility for joint evaluation is defined within the same guidelines describing the procedure for joint assessment in EAC, ECOWAS, and ZaZiBoNa, or are listed in calls for expression of interest (EOI) regularly published by relevant REC secretariats (see Table 1, “Product Eligibility”). African regional JAPs cover a range of essential medicines. However, there is still a considerable variability in eligibility of products depending on the region. Eligibility restrictions can limit patient access to innovative medications and medicines for high burden diseases (11). It also limits the industry’s choice to use JAPs as a pathway to register such medicines or vaccines. In the ARN survey results, participants listed product eligibility as one of the main reasons for not using the respective JAP (see Table 2 and Figure 4).

**Table 2:** ARN survey respondents reporting “product eligibility” as reason for not using respective JAP.

	EAC-MRH	WA-MRH	ZaZiBoNa
Total number of responses	2	9	5
Number of respondents that reported product eligibility as reason for not using respective JAP (%)	2 (100)	4 (44)	2 (40)

ARN Survey Results, June–July 2022. ARN, Africa Regulatory Network; EAC, East African Community; EAC-MRH, East African Community Medicines Regulatory Harmonization; WA-MRH, West Africa Medicines Regulatory Harmonization; JAP, Joint Assessment Procedures.

#### 2.2.2. Adherence to timelines/predictability of the process

The timelines for procedures are defined within the relevant procedural guidelines (see Table 1, “Timelines”). Results from the literature review, as well as the survey, show that in practice applicants are facing delayed approval in comparison to officially defined timelines.

##### 2.2.2.1. EAC-MRH

The reported timelines for the EAC-MRH JAPs are 4–7 months (13). These timelines represent the time to receive a positive outcome of the joint assessment (a recommendation for registration), but not the receipt of the actual national marketing authorizations (MAs), as MAs are still granted by the individual NRAs in a subsequent step.

All applicants reported delays in receiving the national MAs. After providing JAP recommendations to NRAs, it often takes longer than the stipulated 3 months to receive national approval, in some cases more than a year. This led many companies to conclude that the national regulatory approval pathway was faster than the regional JAP, and consequently to prefer the use of the national pathway.

Based on data collected from Mashingia et al. (14), exploring what the EAC-MRH initiative has accomplished in its first 8 years of existence (2012–2020), the median timeline for a joint assessment, from submission of the application to final assessment decision, is just over a year (372 days); from this total amount, 170 days represent the time used by industry to answer queries.

However, the median timeline for a joint assessment in 2019 was only 240 days, indicating that the process has become more efficient in recent years. The national procedure for MA issuance is still longer than the official 90 days. Depending on the country, it may range from 60 to 90 days, 120 to 180 days, and in some cases, longer than 900 days.

**Figure 5:**
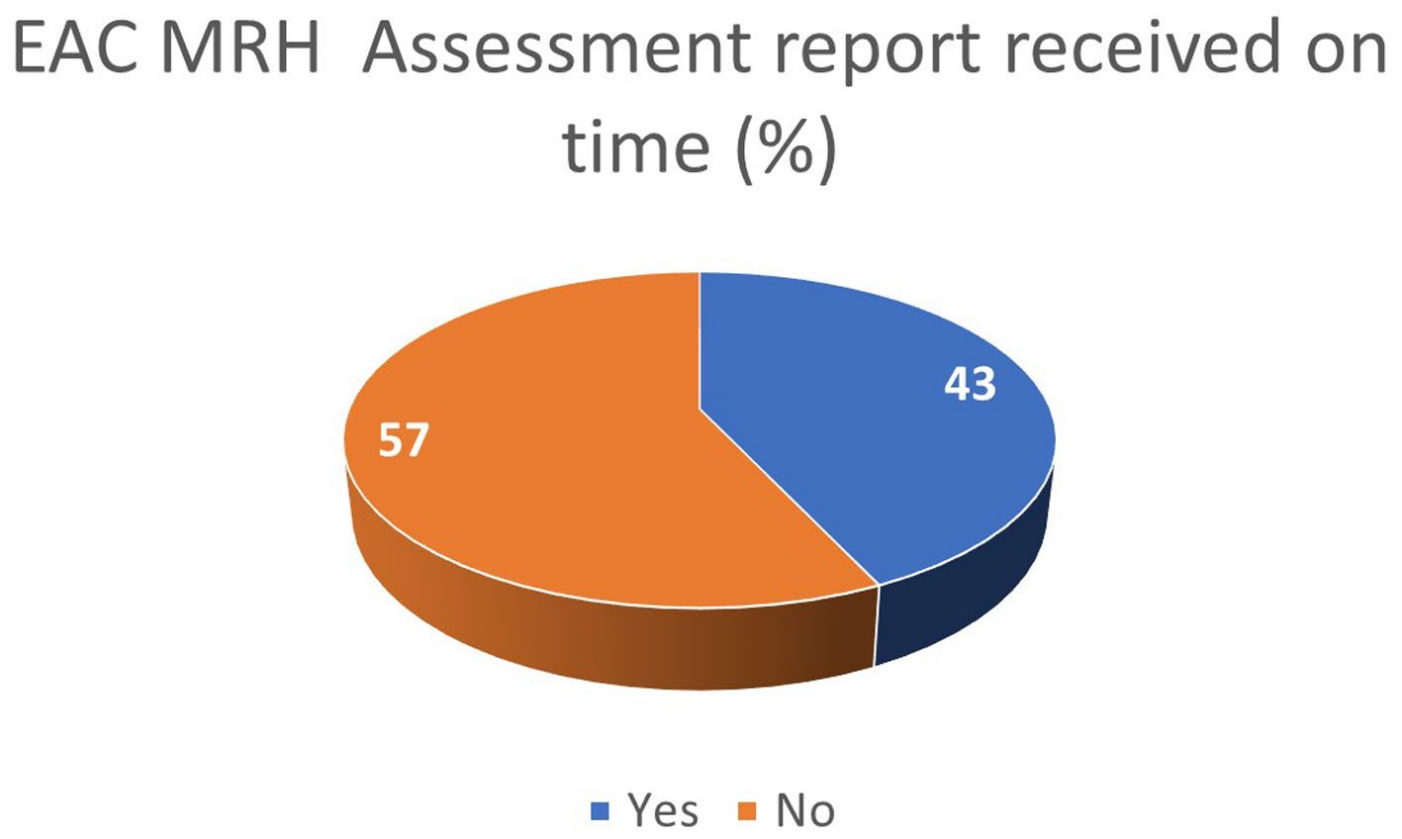
Industry feedback on adherence to EAC MRH timelines.

The results from the ARN survey on the adherence to EAC-MRH timelines show that over 40% of applicants received the EAC-MRH recommendation for registration within official timelines (Figure 5). In addition, as shown in Table 3, 83% of respondents reported that Tanzania was issuing the national MA within official timelines, while delays were reported in other countries from the EAC region.

**Table 3:** EAC-MRH adherence to timelines.

	Yes	No
Did you receive the final EAC-MRH assessment report, including the recommendation for registration, within official timelines (300 days)? (%)	43	57
After the issuance of the final EAC-MRH recommendation for registration, does the individual country issue its national license within official timelines (90 days)?		
Burundi, *n* (%)	17	33
Kenya, *n* (%)	–	100
Rwanda, *n* (%)	–	67
South Sudan, *n* (%)	–	33
Tanzania, *n* (%)	83	17
Uganda, *n* (%)	–	83

ARN Survey Results, June–July 2022. *n* = 11. EAC-MRH, East African Community Medicines Regulatory Harmonization.

##### 2.2.2.2. WA-MRH

There is limited data available regarding to WA-MRH’s procedure timelines, likely because the WA-MRH procedure is the most recent of the three JAPs, being launched only in November 2017, and starting JAP assessments from 2019 (10). The regional guideline foresees 133 calendar days for a complete dossier review with no queries and 196 calendar days with a single round of questions, followed by up to 60 days for NRAs to issue the MA once the West African Health Organization (WAHO) recommendation is submitted.

Indeed, in the ARN survey, we also observed a lower number of respondents, which confirms there are currently less applicants using the procedure compared to the two other regional JAPs.

**Figure 6:**
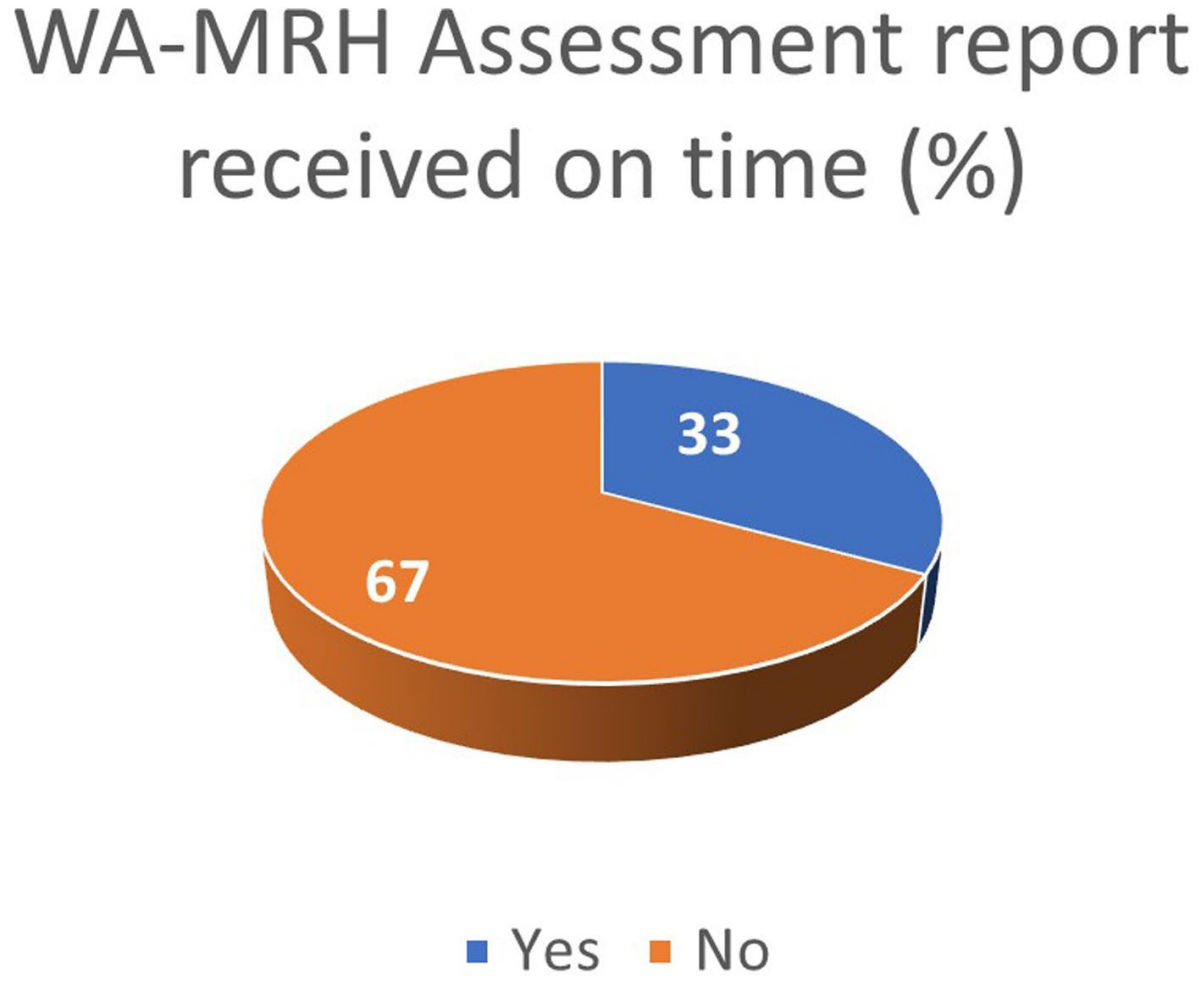
Industry feedback on adherence to WA-MRH timelines.

The ARN survey shows that 33% of applicants received the WA-MRH recommendation for registration within official timelines (Figure 6). In addition, Nigeria and Ivory Coast were reported by some respondents to issue national MA within official timelines.

##### 2.2.2.3. ZaZiBoNa

Approval timelines stipulated in the ZaZiBoNa guidelines are up to 300 days for joint assessment, followed by up to 90 days for MA issuance by participating NRAs.

As reported by Sithole et al., the median time to ZaZiBoNa recommendation, inclusive applicant query response time and excluding individual NRA time before or after joint ZaZiBoNa assessment, varied significantly during the years—from 5 to 18 months in the period from 2014 to 2019 (4).

According to ZaZiBoNa (15), variable data related to approval timelines are reported, with a median ranging from 5 to 18 months.

In the ARN survey, none of the companies obtained a JAP assessment report within the stipulated timeframe. In addition, NRAs’ adoption of ZaZiBoNa’s recommendation varies between countries, showing limited predictability (data available upon request).

#### 2.2.3. Scope of JAPs and impact on lifecycle management

In the relevant guideline documents, specific activities that are under responsibility of the regional assessment are listed (see Table 1, “Scope of the Procedure”). All three regional procedures are working to improve their product assessment procedures. In addition, EAC-MRH and ZaZiBoNa have joint Good Manufacturing Practice (GMP) inspections listed as collaborative activity covered at the regional level. EAC-MRH also includes joint Good Clinical Practice (GCP) inspections, post-marketing surveillance, and safety reporting.

The regulatory oversight of the maintenance of the MAs is an activity of the individual NRAs. They are expected to develop and implement variation guidelines. Applicants are directed to NRA for submission of variations, as well as other aspects related to license maintenance, such as retention and renewal activities.

Lifecycle management (LCM) activities have not been assessed, as they are currently out of scope of the three regional JAPs. However, researchers (7, 16) recommend the inclusion of product LCM and establishment of post-approval change review process to improve the overall approval pathway. Researchers also recommend harmonization of requirements and submission of one application for multiple markets. In the ARN survey, the lack of inclusion of LCM activities was listed as one of the challenges observed in JAPs.

#### 2.2.4. Documentation requirements

In addition to a harmonized Common Technical Document (CTD) or electronic CTD (eCTD) and a cover letter, supplementary documents or samples are required for all three regional procedures (see Table 1, “Requirements”).

It is common for all JAPs to have open provisions that allow participating NRAs to require “any statutory forms and product samples with labeling in compliance with individual country requirements” or allow that “during product evaluation, the NRA may request for further information and additional supporting documents from the applicant” (9). In most instances, mockups of product packages are accepted for assessment. Pre-registration analysis and extensive testing of samples remain a hindrance in some countries, leading to significant time lag between application and MA approval.

Sithole et al. (16) discuss that duplicative requirements are often required to be addressed and provided during the ZaZiBoNa procedure. For example, many WHO specific forms are also used in addition to ZaZiBoNa forms. General national differences of requirement are also a challenge identified by applicants, for example different label requirements and lack of clarity about the submission and follow-up process in each country (16).

Removing country-specific requirements was suggested by both industry (16) and regulators, commenting on the use of ZaZiBoNa as one of the best ways to improve efficiency. According to Sithole et al. (16), authorities in the Southern African Development Community (SADC) region require the submission of the dossier in CTD format; however, there are some country-specific requirements, such as bioequivalence, labeling and local Quality Information Summary (QIS) and Quality Overall Summary (QOS), that still impede harmonization efforts. This is consistent with findings from other studies in the literature (2, 17). Researchers conclude that there is a need for countries to make a deliberate effort to review their legislation to adopt provisions that facilitate the harmonization of the registration and labeling requirements for medicinal products in this region (16).

In the ARN survey, respondents reported that the lack of clarity of the requirements was a major hurdle. Unclear requirements are listed by 66 and 60% of the respondents as the main challenge for not using the WA-MRH and the ZaZiBoNa procedures, respectively. This trend was not recorded in the survey for EAC-MRH.

#### 2.2.5. Clarity of guidance and adherence on regional and national levels

Regional guidelines are available to define regional procedures. However, regional assessment is not the final step of the process, as NRA approval is required to market a product in a country.

In practice, national approval procedures following JAP are not always clearly described in the national guidelines. There are also varying national requirements following the JAP decision (11, 18). Examples include the need to submit the full dossier with application to each of the NRAs following JAP opinion, to provide additional national-specific parts of the application not required with JAP, and to start the national procedure *de novo* (7, 19).

In the ARN survey, divergent requirements at national and regional levels and/or unclear requirements represent a major challenge and a reason for companies deciding not to use WA-MRH or ZaZiBoNa procedures (see Table 4).

**Table 4:** ARN survey respondents reporting divergent and unclear requirements as reason for not using respective JAP.

	WA-MRH	ZaZiBoNa
Total number of responses	9	5
Number of respondents that reported divergent requirements as reason for not using respective JAP (%)	4 (44)	3 (60)
Number of respondents that reported unclear requirements as reason for not using respective JAP (%)	6 (67)	3 (60)

ARN Survey Results, June–July 2022. ARN, Africa Regulatory Network; JAP, Joint Assessment Procedures; WA-MRH, West Africa Medicines Regulatory Harmonization.

#### 2.2.6. Transparency and communication

Clear, understandable, and easily available information for applicants is necessary to efficiently use the JAPs. For example, a published article regarding EAC-MRH (7) has mentioned that the lack of transparent communication and easily accessible information on the procedures hinders its effectiveness and efficacy. The same has been reported for ZaZiBoNa, with one article stating that “although some of the participating countries have information on the ZaZiBoNa on their websites and the contact details of the focal person are known, this is not the case in all the countries and this detracts from the initiative’s effectiveness and efficiency” (4). The same article mentioned that specific and clear requirements made easily available to applicants were identified to contribute to improved efficiency (16).

**Figure 7:**
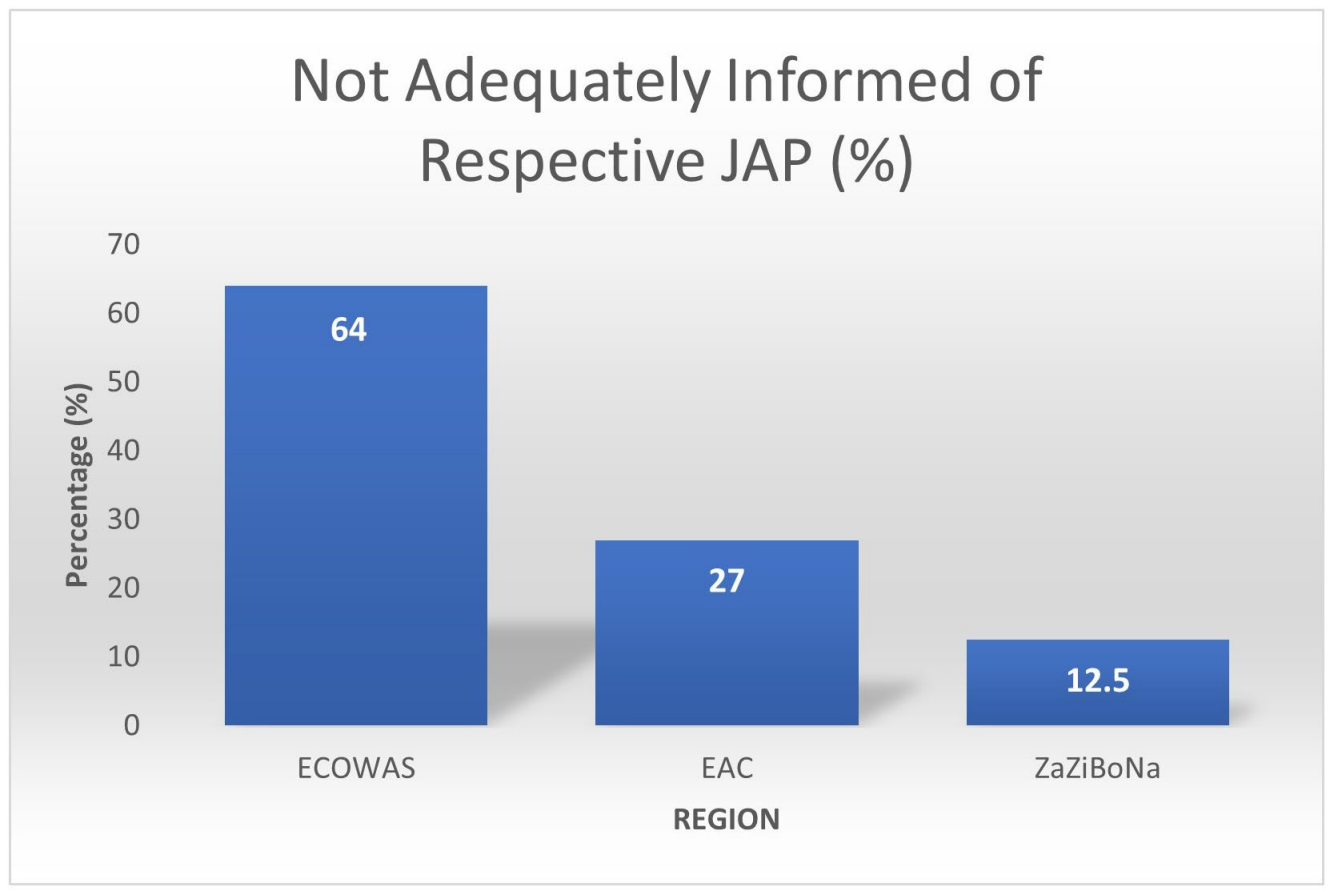
Number of respondents not adequately informed about respective JAP.

In the ARN survey, 12.5% ZaZiBoNa respondents reported they did not feel adequately informed of the procedure. In comparison, 27% EAC respondents and 64% WA-MRH respondents reported the same (Figure 7). Various reasons for the unclarities were listed, including unclear scope, difficulties in accessing information about the procedure online, unavailable timelines, and product eligibility not being indicated. Lack of transparency was reported by 44 and 60% of respondents as one of the main reasons for not using WA-MRH and ZaZiBoNa, respectively.

#### 2.2.7. Appropriate resources

The coordination and follow-up between multiple parties and the applicant during the assessment is challenging to manage. Adequate staffing and resources are necessary to ensure an efficient, sustainable and scalable system (20).

For EAC-MRH, Ngum and colleagues (7) list inadequate human resources as a key challenge identified to hinder efficiency of the procedure. Overall, the need for more assessors to adequately handle the number of applications received was one of the key messages shared. Unequal workload among NRAs was also listed as a challenge, with more well-resourced NRAs finding it much easier to handle applications and queries in comparison to less resourced authorities. Inadequate resources were also reported by both industry (16) and regulators when assessing the ZaZiBoNa procedure. Sustainable funding was also identified as an important prerequisite to ensure a continuously efficient procedure.

#### 2.2.8. Impact of digitalization

The use of digital technologies for data sharing can facilitate collaboration and information sharing. Ngum and colleagues (7) concluded that “the use of a robust information technology system for the central tracking of EAC-MRH products is essential to address the identified challenges and improve regulatory effectiveness and efficiency.”

In reference to ZaZiBoNa, both industry and regulators found that the “inadequate infrastructure and information technology system and resources” were part of the main challenges for a more efficient procedure. In addition, an improved central tracking of applications and a centralized system for the submission of applications and communication with applicants was seen as an opportunity for improved efficiency.

In the ARN survey, the opportunity for digital submission of a dossier was seen as a favorable asset by 57% of EAC-MRH respondents and 33% ZaZiBoNa respondents.

### 2.3. Further regional and global considerations

#### 2.3.1. African Medicines Agency (AMA)

To date (June 2023), 35 of the African Union’s member states have expressed formal support to the AMA Treaty, by signing it, ratifying it, or both. Rwanda has been selected by the Executive Council of the African Union (AU) to host the headquarters of the African Medicines Agency (AMA). It would be important to ensure efficient mechanisms for all the countries on the continent to benefit from the AMA deliverables.

A strong AMRH Governance structure with support of NRAs, RECs and Partners is needed to ensure the operationalization of the continental agency (21) to become one of the most efficient and modern regulatory systems in the world. Driven by the considerable improvements in national and regional procedures achieved during over a decade long harmonization efforts (e.g., with the implementation of AU Model Law in a number of countries), it has potential to foster an encouraging environment for industry and innovation, and contribute to the overall efforts in enabling faster access to medicines, vaccines, and diagnostics in the continent.

In addition, the work of the continental Technical Working Groups towards development of harmonized guidance and procedures, and which are established under the AMRH/AMA umbrella, represents a good basis for a well-organized continental regulatory ecosystem.

Some of the ongoing and upcoming activities in support of AMA include the development of:

- Guidance on priority medicinal products for continental assessment.
- Continental reliance framework.
- Various sets of procedural guidance to facilitate operationalization of AMA and the work of the Technical Committees.
- Comprehensive medicines laws and strong legal frameworks through domestication of the AU Model Law for Medical Products Regulation.

Efforts are also being made to strengthen capacity building (via Regional Centers of Regulatory Excellence - RCOREs), transparency and increase efficiency, through multistakeholder partnerships.

The establishment of the continental regulatory agency needs to be carefully considered in relation to existing national and regional procedures to avoid duplication and redundancy.

**Figure 8:**
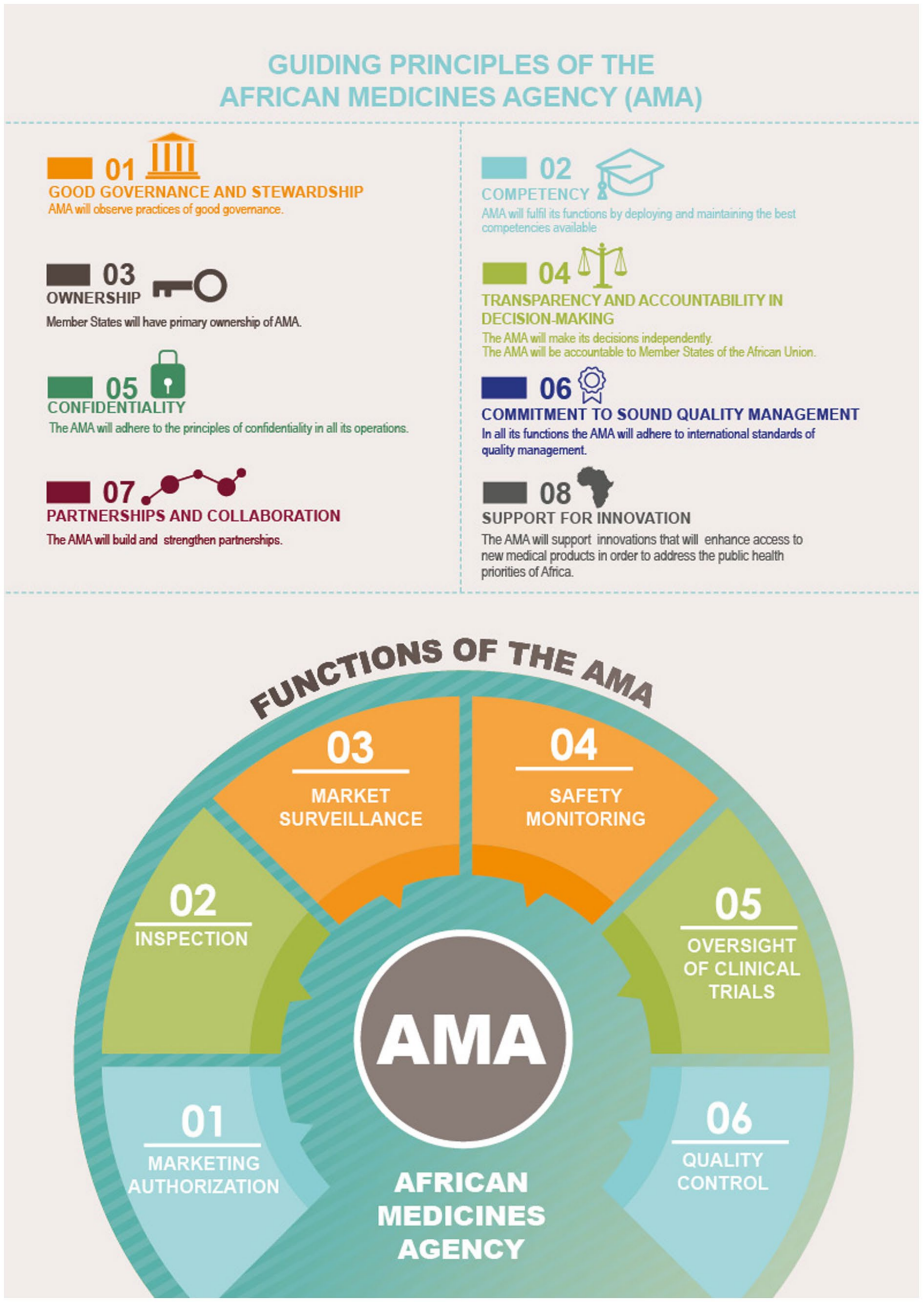
Guiding principles and functions of the AMA. Reproduced with permission from AUDA NEPAD from https://www.nepad.org/publication/ama-inforaphics [Copyright © 2022 African Union Development Agency (AUDA-NEPAD)].

From experiences with other regions (22), a common legal framework is necessary to provide adequate resources and governance structures for a sustainable and efficient system. In that sense, the AMRH and AMA could provide this common umbrella framework for sustainable collaboration, with harmonization of all regulatory activities, spanning the full product lifecycle (Figure 8) (23).

It remains important to clarify how the regional JAPs and future AMA will interact and how the national, regional, and continental roles and responsibilities will be structured and managed to ensure efficient and timely assessments.

It would also be crucial that the AMA framework and the African Continental Free Trade Agreement provide opportunities for more reliance on other regulators’ assessments and extensive work sharing between countries.

#### 2.3.2. Existing collaborative procedures beyond Africa that provide regulatory assessment for medicines in the continent and their correlation to regional JAPs

To support regulatory systems strengthening in developing countries, many well established NRAs (such as EMA and Swissmedic) and WHO have established various collaborative work sharing models to support medicine approval in international regions, including Africa. These pathways offer various advantages to the industry and regulators, including experience sharing and accelerated registration procedures.

Participation in one of the collaborative procedures offers African regulators the opportunity for development and capability building, including knowledge gaining on specific therapeutic areas and on specifics of the assessment of different product categories. It also offers a better understanding on the processes and ways of working in the relevant well-established institution, and some of those learnings could support activities in regulatory systems strengthening on the continent (e.g., EMA experience to support AMA). In addition, this is a good basis for further trust building and facilitation of reliance on the decisions from those well-established institutions going forward.

The following section discusses key considerations related to the various collaborative pathways currently available.

##### 2.3.2.1. EU-Medicines for all (EU-M4ALL)

As part of its contribution to promoting global health, the EMA can assess medicines for use outside of the EU and issue scientific opinions in collaboration with the WHO and non-EU NRAs.

The procedure EU-Medicines for all or ‘EU-M4ALL’, previously known as the Article 58 Procedure, has been in place since 2004. The scope of this procedure includes all medicinal products (i.e., vaccines, biologicals, advanced therapies, small molecules and generics) which are new or improved therapies for unmet needs and diseases of major public health interest, to facilitate patient access in low- and middle-income countries. The scientific review by EMA is informed by WHO program areas and national experts and regulators, to provide a unique development and evaluation pathway (24).

This procedure offers a streamlined process with the possibility of lifecycle management activities. The legal basis in EU law enabling EMA regulators to spend resources for other countries’ benefit and appropriate fee structure also provides assurance (secured resources and system in place) on sustainability of this procedure.

To date, 138 approvals have been granted worldwide in 90 non-EU countries, based on 11 scientific opinions through the ‘EU-M4ALL’ procedure (25, 26). Of the 138, 75 approvals were granted in Africa. ‘EU-M4ALL’ is an effective collaborative pathway improving patient access to medicines in not only Africa, but worldwide. Using the ‘EU-M4ALL’ opinion allows a country to focus its resources on national regulatory areas and helps facilitate complex assessments.

##### 2.3.2.2. Marketing Authorization for Global Health Products (MAGHP) and MAGHP Light Procedure

The MAGHP procedure aims to make the Swissmedic authorization procedure and the scientific advice procedure accessible to representatives of regulatory authorities in low- and middle-income countries, and to the WHO. Although other countries or regions may be involved, the initial focus in the pilot phase is on Sub-Saharan Africa and on medicinal products for those diseases that affect the region disproportionately.

The MAGHP is based on the approach of actively involving regional NRAs and the WHO in the Swissmedic assessment process. The NRAs have the possibility to participate in the assessment with the aim of building their own capacities and establishing confidence in the process. This helps build trust and confidence in the process and is expected to facilitate the granting of national MAs following Swissmedic’s approval. It is expected that the timelines for MA by NRAs will be significantly reduced, accelerating access to essential medicines for patients (27).

The procedure consists of two independent components:

1. Scientific Advice: To clarify scientific questions in the development phase regarding the planned submission.
2. Marketing Authorization:
  - Standard Procedure: the regular Swissmedic marketing authorization procedure, with concerned NRAs and the WHO involved in the process.
  - Light Procedure: special procedure applicable to all applications in the fast track and temporary authorization procedures.

For both the Scientific Advice and the Standard MAGHP Procedure, targeted NRAs actively participate in the process. Active participation implies full access to the applicant’s documentation and active involvement in the procedure. Documents are shared on a secured collaboration platform hosted by Swissmedic.

For the MAGHP Light Procedure, no active interaction is foreseen during the assessment procedure, due to the short and expedited assessment times.

The duration of both procedures follows Swissmedic timelines. In the case of standard MAGHP Procedure, it lasts up to 330 days to obtain Swissmedic’s decision, and then up to 90 days for NRAs to adopt it and issue national MA.

Funding to Swissmedic for this activity is provided by the Swiss Development Agency and Bill and Melinda Gates Foundation.

##### 2.3.2.3. WHO Collaborative Registration Procedure (CRP) for Prequalified (PQ) Products (WHO PQ CRP)

This procedure is applicable to finished pharmaceutical products (FPPs) that are prequalified by WHO (i.e., have been evaluated and inspected according to international standards by a dedicated WHO team). NRAs do not need to carry out full assessments of the prequalified products or to inspect manufacturing sites on their own, but accept assessments by WHO (WHO PQ CRP), thereby enabling faster registration in the country.

Applicants can voluntarily express interest in applying for CRP to their WHO prequalified products. The applicant must submit the same dossier as the one approved by WHO for prequalification, although individual NRAs may agree to submission of simplified dossiers and minor administrative differences are permitted to reflect local labeling and other regulatory requirements. If the NRA agrees to use the procedure, it commits to reaching its decision within 90 days of receiving access to the WHO assessment reports and inspection information. The decision of the NRA must be communicated to WHO and the applicant within a further 30 days.

##### 2.3.2.4. WHO Collaborative Registration Procedure (CRP) for Stringent Regulatory Authority (SRA)-Approved Medicines (SRA CRP)

The WHO CRP is applicable to any SRA-approved FPP relevant to public health needs. Through SRA CRP, NRAs have access to detailed assessment and inspection reports created by SRAs, which are then used for the accelerated registration of the product in their countries. This procedure is based on reliance on SRA decisions. The SRA CRP also adds the component of capacity building for NRA, as NRAs learn from those reports. It requires the exchange of confidentiality agreement letters between all parties.

As of December 2022, the SRA CRP covers 47 participating countries and one REC, as well as seven participating SRAs (28). In order for the procedure to be applied, at least two NRAs should consent to be involved in the collaborative review. A key consideration is that the FPP proposed for registration to participating NRA is the “same” (as defined by the procedure) as the SRA-approved product.

An applicant applies for registration of the FPP(s), with the same set of technical data, electronically and/or in hard copy, to participating NRAs (depending on specific national requirements). Submissions to the different NRA should be made simultaneously.

The timeline for the collaborative review as per the guideline is 90 days from acceptance of the submission by the NRA. The NRA commits to communicate its decision to WHO and the applicant within a further 90 days.

## 3. Actionable recommendations

Regional and global collaborative procedures can offer a wealth of opportunities. The industry should have a clear understanding of each procedure’s benefits and challenges to select the optimal pathway for registration of a specific medicine or vaccine.

**Figure 9:**
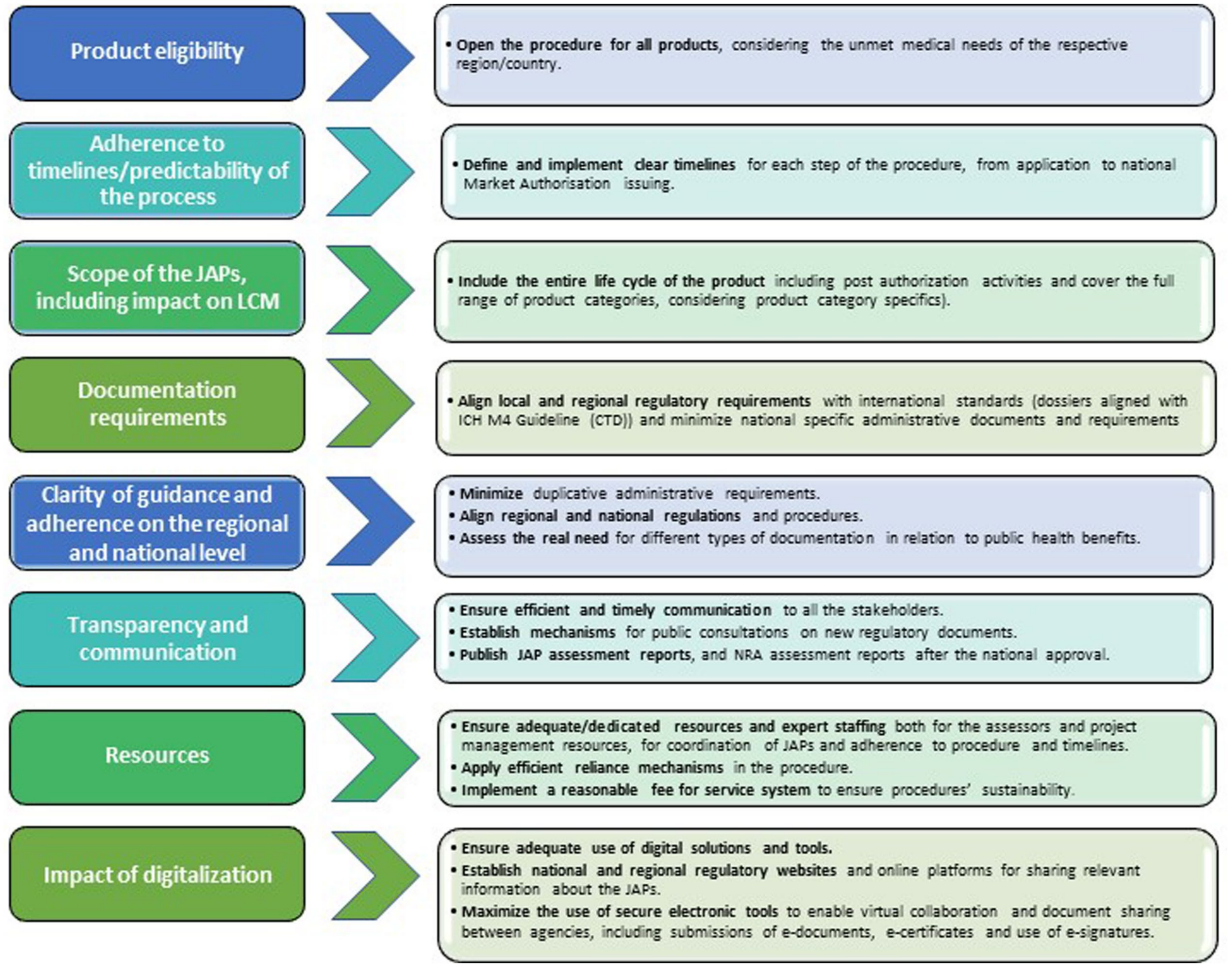
Actionable recommendations for JAPs improvement.

JAPs have the potential to accelerate marketing authorization procedures in the respective regions, which is a first step towards enabling faster access to medicinal products for patients. However, the regional JAPs could be further improved by the following recommendations listed below (Figure 9).

### 3.1. Product eligibility

Regional JAP eligibility criteria should be further harmonized to increase the scope of products of interest (e.g., neglected diseases, innovative medicines and vaccines) and to optimize the use of JAPs. Ideally, all medicinal products should be eligible, considering the unmet medical need of the respective region/country. In addition, there should be a mechanism in place to continuously update the list of eligible products in line with evolving needs by the regions/countries.

### 3.2. Adherence to timelines/predictability of the process

Clear timelines for each step of the procedure need to be defined and implemented. Adherence to these timelines is essential for a predictable and attractive procedure. JAPs have led to a general reduction in review timelines (7) in comparison to national procedures. Defining timelines on when to expect questions to applicants are also important to plan adequate resources for responding, which can shorten the time needed by applicants. It is also important that industry commits to responding to the questions raised during the joint review process in a timely manner to ensure a reduction in clock stop time. Besides timelines for the joint assessment process, there should be firm commitment from participating NRAs to ensure that the final step for marketing authorization issuing is not delayed and that it is fulfilled within the set timelines by individual countries.

### 3.3. Scope of JAPs and impact on Lifecycle Management (LCM)

Regulations should cover all activities throughout the product lifecycle to ensure continued access to medicinal products for patients in Africa. In addition to efficient new product registration processes, fast and efficient post-approval changes (PACs) procedures are essential to avoid unintended disruptions to continuous supply of medicines and vaccines.

The scope of regional collaborative activities should be expanded to cover all post authorization activities, such as PACs, renewals and retentions, pharmacovigilance, and market surveillance.

The JAP guidelines should cover the full range of product categories including small molecules, biotherapeutic products and vaccines, taking into consideration product category specifics.

### 3.4. Document requirements

Lot of progress has been achieved through the implementation of international standards [e.g., International Council for Harmonization (ICH), WHO guidance, and GMPs according to Pharmaceutical Inspection Co-operative Scheme (PIC/S) standards] and the development of common regional procedures. The harmonization of regulatory requirements is the foundation for joint assessment (29).

Fully harmonized dossiers according to ICH M4 guideline (Common Technical Document) should be accepted by all NRAs participating in the JAPs, with no additional national requirements. Specific national requirements, such as the request for physical product samples, should be adapted or waived (i.e., acceptance of mockups) to speed up submissions. Such practice has already been adopted by mature regulatory systems many years ago.

Our assessment shows that there are still many specific national requirements that must be fulfilled for local and regional submissions. Some examples include the requirement for legalization of specific documents, or national specific forms and declarations needed for application.

Different formats for submission between NRAs (e.g., paper copies in some NRAs, digital or on-line platforms in another) in different JAPs also pose a challenge when submitting a product in different African regions. This leads to more dossier versions, while potentially delaying dossier preparations and submissions to multiple countries.

We recognize that any joint assessment outcomes must always be considered within the local healthcare system context. While scientific and healthcare system specificities are important, purely administrative issues should be minimized.

Therefore, fully aligning local and regional regulatory requirements with international standards and minimizing national specific administrative documents helps applicants in preparing the same global core dossier for use in joint assessments. A common guideline would also help streamlining and accelerating joint post-approval change management, avoiding delays that may affect continuity of supply in countries.

### 3.5. Clarity of guidance and adherence on regional and national levels

Administrative burden should be minimized, and the focus must be on ensuring appropriate quality, safety and efficacy of a product. When unnecessary administrative requirements are requested by national legislation, countries and NRAs should consider taking steps towards initiating legislative changes to remove such unnecessary burden. Alignment and consistency between regional and national regulations and procedures is a must.

Practical considerations include:

- Avoiding duplication of submissions at national level.
- Waiving pre-registration sample testing requirements.
- The establishment and use of a common digital submission portal with secure access for participating NRAs to support better coordination.
- Consolidated list of critical questions during the JAP and no additional questions to this assessment during the national step.
- Clear pathway for swift national approval after JAP assessment, defined both in the national regulations and in JAP guidelines and standard operating procedures (SOPs).

Regulations at the national level that describe the regional JAPs and the national adoption of joint assessment opinions would help clarify the overall process to avoid complexity and delays observed. Without such comprehensive guidance, the process is left to individual NRA interpretation and case-by-case handling of applications. We suggest establishing regulated and institutionalized processes at regional level, but also integrating these into national regulations and NRA’s procedures. It would also be helpful to clarify the connection between JAPs and NRA procedures.

A specific national procedure, separate from the conventional national pathway, to transpose JAP outcomes into national decisions, should be included into national legislation and guidance.

This should be complemented by clear NRAs’ operating procedures and dedicated resources to issue a national decision within stipulated timelines. This could also be supported by having dedicated in-country coordination personnel responsible for coordination of approvals for products assessed through the collaborative pathway.

Tracking and publication of approval timelines would provide increased accountability and transparency to the public.

Requirements for documentation are not always aligned on the regional and on the national levels (13). Redundant content is often provided via similar but not identical templates, which is inefficient and resource intensive. Also, the use of common templates (e.g., WHO) for the joint procedure, instead of country specific templates, would help avoid duplication of efforts. Harmonization of requirements at national and regional level or waiver of certain country specific requirements for products that have been assessed following a joint review would also ensure faster approval at country level. The simpler and more harmonized the procedures across the regions are, the easier it would be to follow them, and the more likely industry will use them.

It would be advisable to assess the real need for different types of documentation in relation to public health benefits and clarify their relevance. Requirements should be based on a clear rationale with no redundant or duplicative documents.

At this time, the different product types are not always treated differently. The procedures and requirements for products need to be tailored to the type of products assessed because of their specificity and the different risk they pose. Risk-based procedures for more complex treatment therapies, such as advanced therapies medicinal products (ATMPs) or specific combination products must be considered.

### 3.6. Transparency and communication

Collaboration among stakeholders, including regulators and industry, to build trust and enable transparent communication is key. This will facilitate the implementation of regulatory reliance practices (30) and could accelerate NRA approvals.

We recommend publishing JAP assessment reports after the opinion and NRA assessment reports after the national approval to increase accountability and transparency in the process. This best practice could also help to use JAP assessment outcomes across multiple regions.

Industry is encouraging regulators to share relevant documents among themselves in a secure and structured manner (29). The increased utilization of Public Assessment Reports (PARs) (31) would help facilitating initial decision-making, stimulate interactions among JAPs, facilitate the use of resources and enable reliance across regions.

Information necessary for efficient procedures (timelines, schedules of activities, expectations for queries, etc.) should be publicly available to ensure users can easily access these resources for their regulatory strategy planning. Resources of both regulators and the industry are limited. Predictable procedural timelines would significantly help applicants in appropriate resource planning and allocation to provide timely and adequate responses to questions. Furthermore, earlier information on timings of planned assessment meetings would help planning of application submissions. This will ensure the earliest possible submissions to facilitate an earlier availability of therapies to patients.

### 3.7. Appropriate resources

The adequate allocation of resources, both human and technical, is a prerequisite for successful JAPs. RECs are encouraged to continue developing and implementing sustainable operating models that are scalable and can handle a larger volume of applications (20). Both expert resources and dedicated project management resources for coordination of JAPs are required to ensure adherence to timelines.

The coordination and follow-up with multiple parties and the applicant during the JAP is not easy to manage, therefore adequate staffing for project management is key to ensure a sustainable and scalable system.

Close collaboration between countries is required to ensure an adequate and shared expert pool, especially if the staffing is managed locally. Dedicated expertise for different therapeutic modalities, like small molecules and biotherapeutics, would be beneficial.

Risk-based approach and more reliance in the area of GMP inspections (32) and on assessments by selected reference health authorities such as SRAs or WHO PQ, could support efficient use of resources for all participants. A predictable process with limited number of queries and no requests for additional data to be generated during the assessment process (in addition to ICH requirements) could help addressing some resource bottlenecks.

A sustainable funding system to ensure adequate support is also essential. Indeed, better adherence to registration timelines was seen in regions where there were dedicated financial resources for managing collaborative efforts (33).

A fee for service system that can guarantee the efficient timelines of JAPs could be established (34). Such approaches have worked well in Europe (35) and the United States (36).

A fee for service system, with defined KPIs and roles and responsibilities, that is implemented on national, regional, and continental level, could provide additional incentives for participating countries, allowing JAPs and NRAs to receive a proportionate amount of the commonly collected fee for their service. Incentives for NRAs should be established to mobilize national resources on supra-national level, in order to prioritize and perform this work. Fixing clear Key Performance Indicators (KPIs) to fee for service scheme would encourage applicants to use a certain pathway.

Sufficient resources to perform the administrative coordination task among participating countries, including a digital working environment to facilitate collaboration and communication, must be available and sustainable funded.

In the longer term, available national resources should be allocated to value-adding national tasks, which could free up resources for regional or continental activities. AMA could be seen as a potential solution to address the challenge related to resources and funding. This would imply the revision of existing provisions of AMA Treaty and relevant legal frameworks, allowing decision making power to the continental agency.

### 3.8. Impact on digitalization

The development, implementation, and maintenance of enhanced information communication technology (ICT) solutions to facilitate accurate tracking of applications, decision making, document management, transparency, and stakeholder communication are needed. This should be supported by dedicated resources.

The increased use of digital technologies during the COVID-19 pandemic was successful in accelerating the regulatory process, allowing the reduction in administrative burden and thus a more efficient use of limited regulatory resources. Some regions have already established digital platforms for submission and information sharing. However, there is a need to continue and broaden the implementation of these tools. Further work on harmonization of submission formats and implementation of ICH eCTD (M8 guideline) would be beneficial.

Digitalization is a long-term project, which can be gradually applied in the current way of working by:

1. Increasing the use of national and regional regulatory websites and online platforms, supporting transparent and easy access to JAP guidelines, detailing procedural requirements and timelines.
2. Facilitating the exchange of feedback during public consultations on regulatory documents or at the publication of new regulatory guidance and information on medicines regulation.
3. Maximizing the use of electronic platforms, e-communications, e-documents and virtual work sharing.
4. Minimizing the administrative burden and delays by using electronic Certificates of Pharmaceutical Product (eCPP) when relevant, e-signatures and acceptance of databases such as EudraGMDP (37).

Longer-term digital transformation to enable strong regulatory framework should consider developing secure work-sharing tools that allow simultaneous review and feedback (e.g., SharePoint, cloud-based platforms/solutions), and harmonized dossier formats (e.g., eCTD).

## 4. Discussion

This article assessed existing registration pathways to license medicinal products in African countries, with the special focus on regional JAPs. It reviewed the experience in the context of AMRH initiative and the global regulatory science evolution. Our aim was to identify the benefits of each existing pathway and to provide recommendations for collective improvements, allowing the industry to make informed decisions when planning their filing strategies for the region.

The ongoing harmonization activities and convergence of standards are the basis for the collaboration among regulators and for work-sharing and reliance, leading to a more efficient use of resources and offering the opportunity for faster product registrations. It is specifically important to further clarify the scope and responsibilities of the three regulatory layers in the African regulatory ecosystem—(national, regional and continental), to avoid duplication and redundancy.

With the established regional JAPs in Africa, we observed the following positive aspects, and would like to encourage all stakeholders to continue the good progress in those areas:

- Shorter timelines for joint product assessment (not including issuance of national marketing authorization).
- Generally predicable appointment/assessment meetings.
- Flexibility to also assess products not included in the list of eligible products on a case-by-case basis.

It would be important to make above mentioned aspects even more consistent and sustainable at regional level by adding appropriate provisions to official guidance and procedures and implementing them nationally.

Areas for further collective improvement include:

- Better coordination of roles between RECs and NRAs.
- Allocated point of contact, available for communication and process follow up.
- Predictable and consistent time to the national marketing authorization issuing.
- Minimization of country-specific requirements, in addition to regional JAP-specific requirements (e.g., pre-registration analysis, physical samples, additional declarations or forms), leading to different dossiers among countries and complexity in lifecycle management activities, which further affects continuous supply.
- Streamlining of administrative challenges, such as variable and complex submission procedures, and less frequent assessment sessions leading to delays.
- Broadening the scope of the JAPs to the whole product lifecycle.
- Awareness and utilization of JAPs by industry.

Potential solutions that could aid to improving identified challenges include:

- More harmonization of requirements across countries and regions, assessment of the public health value of national specific requirements, and adequate adjustments.
- Definition of a clear pathway for national approval by setting administrative expectations for NRAs participating in JAPs.
- Adequate “fee for service” mechanisms to be put in place in order to ensure the sustainability of a continental system, in consultation with all the stakeholders including industry.
- Adequate resource allocation for JAP activities, and ensuring adherence to timelines and requirements (e.g., through fee for service mechanisms).
- JAPs applicability to the entire lifecycle of the product (i.e., products initially approved via JAP should also have all LCM activities handled through JAP).
- Better use of regulatory reliance amongst NRAs to allow a reduction in queries and faster review.
- Use of digital technologies for data sharing to facilitate collaboration and information sharing, and ensure patient involvement in treatment decisions and outcomes.
- Transparency in the decision-making process, through the publication of assessment reports that could be reused by other regions and further add to efficient use of reliance.
- Improved communication and awareness of the benefits of the JAP procedures among applicants via infographics jointly developed between industry and regional programs (e.g., EAC-MRH, WA-MRH) (38, 39).
- Regular dialogue on the JAPs (success stories, experiences) between regulators and industry to improve the process and increase its utilization by applicants.

We have noticed that some regions have already acknowledged some of the points raised above and are working towards their improvement (e.g., ZaZiBoNa/SADC working on the development of common procedures for variations handling, proposals for introducing sustainable financing models in EAC and ZaZiBoNa, organization of regional stakeholder meetings and consultations to develop proposals for JAP improvements, etc.). Though this is still a work in progress, it is a good step in the right direction.

The industry was involved in the use of the JAPs since their inception (initial pilots). This engagement was a good opportunity for industry and regulators to collaborate and learn together. We encourage a more systematic approach in engaging the industry early enough in all the regulatory processes to be put in place in the continent, for a successful establishment of a robust African regulatory ecosystem. The industry is willing to continue partnering with other stakeholders to strengthen regulatory systems and improve the established procedures which will ultimately improve/facilitate access to medicinal products to all patients in Africa.

## Author contributions

NM, SA, AJ, JA, JM, and ZA contributed to the conception and design of the surveys and manuscript. SA developed surveys, collected, and analyzed the data. NM wrote the first draft of the manuscript. Tables and figures were prepared by SA and NM. All authors contributed to the article and approved the submitted version.

## Conflict of interest

NM is employed by F. Hoffmann-La Roche. JA is employed by Johnson & Johnson. JM is employed by Bayer Pharmaceuticals. ZA is employed by Novartis. AJ is employed by MSD.

The remaining author declares that the research was conducted in the absence of any commercial or financial relationships that could be construed as a potential conflict of interest.

## Publisher’s note

All claims expressed in this article are solely those of the authors and do not necessarily represent those of their affiliated organizations, or those of the publisher, the editors and the reviewers. Any product that may be evaluated in this article, or claim that may be made by its manufacturer, is not guaranteed or endorsed by the publisher.
